# Rhizome of life, catastrophes, sequence exchanges, gene creations, and giant viruses: how microbial genomics challenges Darwin

**DOI:** 10.3389/fcimb.2012.00113

**Published:** 2012-08-28

**Authors:** Vicky Merhej, Didier Raoult

**Affiliations:** URMITE, UM63, CNRS 7278, IRD 198, INSERM U1095, Aix Marseille UniversitéMarseille, France

**Keywords:** catastrophes, Darwin, gene creation, giant viruses, micorbial genomics, rhizome of life, sequence exchange

## Abstract

Darwin's theory about the evolution of species has been the object of considerable dispute. In this review, we have described seven key principles in Darwin's book *The Origin of Species* and tried to present how genomics challenge each of these concepts and improve our knowledge about evolution. Darwin believed that species evolution consists on a positive directional selection ensuring the “survival of the fittest.” The most developed state of the species is characterized by increasing complexity. Darwin proposed the theory of “descent with modification” according to which all species evolve from a single common ancestor through a gradual process of small modification of their vertical inheritance. Finally, the process of evolution can be depicted in the form of a tree. However, microbial genomics showed that evolution is better described as the “biological changes over time.” The mode of change is not unidirectional and does not necessarily favors advantageous mutations to increase fitness it is rather subject to random selection as a result of catastrophic stochastic processes. Complexity is not necessarily the completion of development: several complex organisms have gone extinct and many microbes including bacteria with intracellular lifestyle have streamlined highly effective genomes. Genomes evolve through large events of gene deletions, duplications, insertions, and genomes rearrangements rather than a gradual adaptative process. Genomes are dynamic and chimeric entities with gene repertoires that result from vertical and horizontal acquisitions as well as *de novo* gene creation. The chimeric character of microbial genomes excludes the possibility of finding a single common ancestor for all the genes recorded currently. Genomes are collections of genes with different evolutionary histories that cannot be represented by a single tree of life (TOL). A forest, a network or a rhizome of life may be more accurate to represent evolutionary relationships among species.

## Introduction

The theory of evolution became a subject of deep reflection toward the end of the twentieth century. The development of the theory of evolution has benefited from the contributions of several authors, including Lamarck and Darwin (Koonin and Wolf, 2009). Their findings have been subjected to intense criticism. Indeed, their claim that all living species were transformed over time to give rise to new species was much to the dismay of the creationists (the equivalent of the “fixistes” in France) who believed that each species was created once and for all and that no species had disappeared since the creation. This latter perception of the worlds is a synthesis between the Socratic Greek philosophy, the harmonious cosmos and the essentialism of Plato (427–327 BCE) and Aristotle (384–322 BCE) on one hand and the Christians' view of the world's creation as described in the bible on the other hand. In contrast, the monistic view of Heraclitus (535–475 BCE), the constant motion of Democritus (460–370 BCE) and the dynamic theory of atomic motion described by Lucretius (94?–55 BCE) considered life to be an interplay of physical-chemical forces immanent to matter and in which living things live in perpetual motion. In this context, Lucretius' Epicurean poem, *De rerum natura*, postulated the extinction of species that are not well suited to surviving and reproducing successfully (Lucretius, 1995).

Darwin developed a highly disputed theory that was largely influenced by the works of Buffon on transformism (de Buffon, 1753), the concept of the differential fertility of Malthus (Malthus, 1798; Barlow, 1958) and the gradualism of Leibniz (Leibniz, 1996). Darwin proposed a straightforward mechanism of evolution that involves an interplay between heritable variation and natural selection, collectively described as the survival of the fittest. Under Darwin's concept, the material for evolution is provided by heritable random variation; natural selection is the main driving force of evolution, which introduces order and produces increasingly complex adaptive features of organisms. Darwin thought of natural selection in terms of the fixation of beneficial changes, i.e., evolutionarily relevant mutations. These beneficial changes have infinitesimally small effects on fitness, and, as a result, evolution occurs via numerous, successive and slight modifications according to the theory of *strict gradualism*. Finally, Darwin suggested that all life forms evolved from a single common ancestor (Darwin, 1859). Indeed, based on his observations on the evolution of animals, Darwin attempted to issue a general theory about the evolution of life. He proposed that the relationships among all species resemble a tree, the Tree of Life (TOL), in which all living organisms are considered to have descended from a single ancestor (Darwin, 1859).

Darwin's theory was later the object of considerable dispute, particularly because Darwin was unaware of Mendel's work and of the importance of genetics for understanding evolution (Charlesworth and Charlesworth, 2009). Fisher, Haldane, Dobzhansky, Wright and Mayr, among many others, integrated genetics, paleontology, systematics, and cytology within a newly expanded structure of biological thought that is often referred to as “the modern Synthesis” (Huxley, 1942; Koonin, 2009d). The modern synthesis provided useful foundations for biological thought, including the idea that changes in genotype, the genetic material, precede changes in the phenotype, which determines the appearance of an individual. The modern synthesis framework provided many fundamental insights into evolutionary biology, especially with regards to the main topic of Darwin's famous book, *The Origin of Species* (Darwin, 1859). Darwin thought that species were the result of the human predilection to perceive discontinuity among continuously varying individuals. Mayr's extensive knowledge about variation in morphology, overlain with an understanding of the biogeographic distributions of bird species, led him to develop the biological species concept. Mayr explained the geographic mechanisms of speciation and insisted that the geographic separation of populations that prohibits a homogenizing gene flow between them leads to the divergence of such populations and to reproductive isolation. Based on these concepts, Mayr defined allopatric speciation as, the process of the evolution of geographically isolated populations into distinct species, and sympatric speciation as, the evolution of new species inhabiting the same geographic region (Mayr, 1944, 1963).

In this paper we outline the changes brought about by comparative genomics and phylogenetic studies as determined by Koonin to the concepts of evolution proposed by Darwin (Koonin, 2009a) (Table 1). We have identified seven key principles in *The Origin of Species* (Darwin, 1859): (1) the concept of evolution according to a positive directional selection that favors advantageous mutations to increase fitness, (2) the struggle for existence, (3) the complexity associated with development, (4) gradualism and progressive evolution, (5) strict vertical inheritance, (6) a single common ancestor, and (7) the TOL. We discuss these points to identify the hypotheses that survive critical analysis and respect current knowledge. We attempt to highlight the influence of microbial genomics on our understanding of the evolution of genetic repertoires.

**Table 1:** **Darwin's propositions in the face of evolutionary genomics**.

**Darwin's proposition**	**Genomic challenge**
The general trend of evolution is the fixation of beneficial changes	Natural selection is one of the evolutionary forces. However, random selection is largely produced by catastrophic stochastic processes
According to the principle of the “survival of the fittest,” organisms evolve toward the most well-adapted state	Genomes contain many genes that do not increase the fitness and genes that are not required for the survival in current ecosystems
The general trend of evolution leads to complex adaptive organisms	Complex organisms represent very small part of living species. Several complex organisms have completely disappeared
Organisms evolve through the gradual fixation of infinitesimally small variations by natural selection	Genomes evolve through large events of gene deletions and duplications and insertions and genomes rearrangements. Evolution rarely follows a gradual adaptative process
Organisms evolve through vertical inheritance of ancestral characters	The gene repertoire results from vertical and horizontal acquisitions as well as *de novo* gene creation
All cellular life forms have one common ancestor	The chimeric character of the genomes excludes the possibility of finding a single common ancestor for all the genes recorded currently
The evolution of life can be depicted as a single tree (TOL)	Genomes are collections of genes with different evolutionary histories that cannot be represented by a single tree of life

## Natural selection

The question of our origins has always fascinated humans. From the earliest times, the existence of life has typically been attributed to supernatural intervention. Naturalistic models of origins based on logic and philosophy can be traced to approximately the fifth century BC in Greece at the time of the pre-Socratic philosophers and scientists (Anaximander, Heraclitus, Empedocles, Parmenides, Zeno, Democritus…). Anaximander argued that life originated in the sea and deduced living beings gradually developed, from moisture and warmth. He further proposed that the first human, in the form known today, originated from animals of another sort (Barnes, 1983). Empedocles claimed that living creatures might have originated by chance (Barnes, 1983). In contrast, the process of development was denied by the philosophers Plato and Aristotle. These philosophers denied any continuous change of ideas or forms, i.e., the forms, or archetypal ideas, remain eternally what they are. Thus, evolution was considered by Plato and Aristotle to be a general trend in which everything in nature has a certain order or purpose. The physical world is wholly dominated by purpose (Aristotle, 2008). Aristotle developed a “*scala* of *naturae*,” a great chain of being, in which he arranged all beings on a ladder beginning with inanimate matter and climbing to plants, invertebrates, and vertebrates. Among the vertebrates, Aristotle placed the fish at the lowest rung of the ladder and humans on the highest rung. This scale of nature is a graded scale of perfection that represented a continual progression from simple and undeveloped organisms to the complex and more perfect organisms (Singer, 1931; Mayr, 1982).

Darwin was not the first to describe the origin of species as one from another as a formal doctrine. In addition to the Greeks mentioned above, Lamarck denied the immutability of species and forms and claimed to have demonstrated by observation the gradual development of the animal kingdom (Lamarck, 1809). What is new in Charles Darwin's work is not his theory of descent but its confirmation by the theory of natural selection and the survival of the fittest in the struggle for existence. The major contribution of Darwin to the idea of selection can be summarized with the words “chance and necessity.” According to Darwin, changes in the genome occur by chance and are maintained if necessary (natural selection). The resulting genomic repertoire corresponds to a rational end in a purely mechanical process without any cooperation of teleological principles and without any innate tendency of the organisms to proceed to a higher stage. This theory postulates that the later organisms deviated from the earlier ones and that these deviations, in so far as they are improvements, perpetuate themselves, and become generic marks of differentiation. Interestingly, the words “chance and necessity” were used for the first time by Democritus, who ascribed the causes of things either to necessity or to chance and the absence of purpose (Barnes, 1983). Democritus showed that apparently orderly effects can be produced without goal-oriented forces or purpose. Nietzsche prone the realm of chance “Those iron hands of necessity which shake the dice-box of chance, lay their game for an infinite length of time …” (Nietzsche, 2006). Similarly, Jacques Monod [winner of the Nobel Prize in Physiology or Medicine (1965)], in his famous book “*Chance and Necessity*” (Monod, 1972), described the structural teleonomy of living organisms with apparent intended goals and refuted the idea of purpose in nature.

Charles Lyell, a famed geologist and paleontologist, befriended Charles Darwin and strongly influenced his thought. Lyell's interpretation of geologic change prompted Darwin to think of evolution as a slow process in which small changes gradually accumulate over immense spans of time. Lyell had shown how gigantic valleys had been formed by gradual erosion; similarly, Darwin believed that natural selection occurred through the preservation and accumulation of a great number of infinitesimally small inherited modifications (Lyell, 1830). This theory intentionally ignored the catastrophic (chaotic) events (such as earthquakes) that the creationists used to explain evolution and the presence of fossils by defining fossils as living beings that coexisted with current living beings but that had disappeared under the impact of a disaster. These events drastically reduced population size and resulted in genetic drift. Indeed, more than 99% of a population might be killed by disasters, allowing only a few genetic features to be selected. This random selection occurs frequently in microbiology especially in the digestive microbiota. The invasion of a new bacterial or a viral species can cause diarrhea, which can lead to the extermination of up to 10^13^ bacteria, archaea and bacteriophages; the use of a specific antibiotic treatment, such as metromidazole, can eradicate 90% of the population in a few days. Interestingly, these ecosystems are repopulated at considerable speed and contain new species particularly in the presence of antibiotics that prevent the revival of the original flora. Thus, we can observe the effect of disasters on microbial populations, and there is no reason the same types of disasters, less common but just as critical for evolution, have not affected all living things.

When considering the important role of catastrophic events in the selection of living beings, evolution more closely resembles a random process than a mechanism driving positive selection. Recent work particularly that of Abi Rached et al., has shown that humans are a mosaic of three currently known hominids: Cro Magnon, Denisovan and Neanderthal. It is likely that following a series of catastrophic accidents, some mixed populations survived in different parts of the world (Abi-Rached et al., 2011). Horses evolved between 54 million years ago to about 10,000 years ago, spreading throughout North America. Then, suddenly, without apparent reason, between 10,000 and 8000 years ago, *Equus* disappeared from North America. Various theories have been advanced including destruction by drought, disease, or extinction as a result of hunting by growing human populations. At any rate, the horse was gone, and the horse was not seen again on its native continent until the Spanish explorers brought horses by ship in the sixteenth century. In total, the elements that create a visible disaster bottleneck are likely key to the selection of species (Remington, 1889).

## The struggle for existence

Darwin believed that each organism must fight for its existence to survive, as derived from Malthus' theory on the population (Malthus, 1798). Darwin noted that each generation tends to increase, a process that should produce an overabundance of beings in nature. However, space and food are limited. The permanent destruction of some living beings is therefore necessary because not every individual can survive; this results in fierce competition among individuals of the same species and among individuals of different species. Thus, the struggle for existence arises from the inherent limitations of an ecological environment and the increasing number of species. Natural selection is the result of a struggle for existence, what Darwin called “the survival of the fittest” (Darwin, 1859). Natural selection eliminates some lineages and supports the species best adapted to their environments. Favorable variations in terms of survival and reproduction tend to be preserved, and unfavorable ones are destroyed. In contrast to the well-accepted statement of Lucretius about the necessity of reproduction for a species to endure (Lucretius, 1995), the notion of a struggle for existence remains a debatable issue. Indeed, some mutations may alter the fitness without decreasing the ability to multiply and perpetuate.

According to Darwin, organisms always evolve toward the most well-adapted state; thus, nearly all components of the genome should have a beneficial function. However, in microbial organisms, the gene content raises questions about the principle of the survival of the fittest. Bacterial species have a number of genes in common that compose the core genome, and partially shared and strain-specific genes; the total of these genes constitutes the pan-genome (Tettelin et al., 2005; Schoen et al., 2008). Some bacteria that live in an ecosystem with highly variable conditions and with many other bacteria (sympatric bacteria), such as *Escherichia coli* or *Pseudomonas aeruginosa*, have very broad pan-genomes. Other bacteria that live in ecosystems with very restricted physicochemical conditions and limited partners (living in allopatry) have much smaller pan-genomes (Moliner et al., 2010). Interestingly, sympatric species retain some unused genes that are expressed at low rates. This pool of genes is not required for survival in the current ecosystem but may become necessary after future changes in the ecosystem. Indeed, bacteria contain laterally transferred sequences of DNA that are generally nearly neutral to the recipient and exert no effect on its fitness (Gogarten and Townsend, 2005). Much of the bacterial genomes consists of selfish elements with no appreciable phenotypic effect and that function only to ensure their own self-preservation within the genome (Orgel et al., 1980; Ogata et al., 2000).

**Figure 1:**
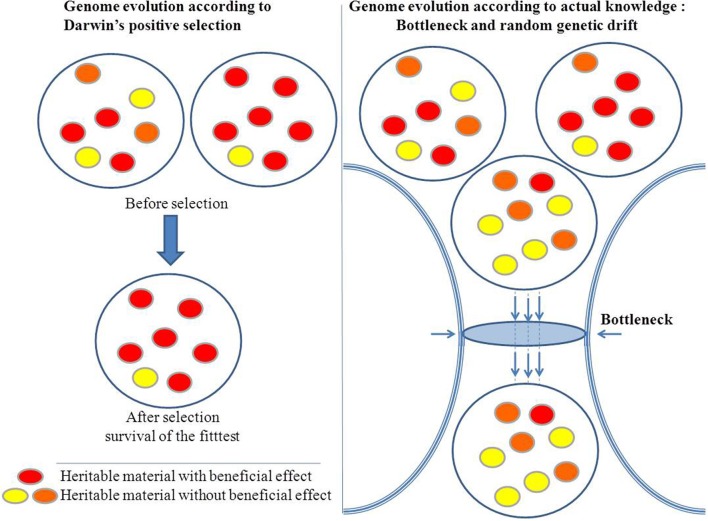
**Natural selection versus stochastic processes**.

Evolution as described by Darwin is a process of unidirectional positive selection that favors advantageous variations and results in increased fitness. In microbiology, mutations in DNA gyrase and in RNA polymerase that confer resistance to antibiotics such as quinolones or rifampicin may allow a bacterium to persist in its environment and, thus, seems to illustrate the Darwinian adaptive evolution. However, in most cases, these changes are not accompanied by an increase in fitness, and the mutants are rapidly eliminated when the antibiotics are removed. Hence, the change is purely opportunistic and does not play a role in the long term. Indeed, as for antibiotic selection, the antibiotic resistance of a microorganism may be associated with a short term advantage and with loss of fitness at long term when the ecosystem changes (with no more antiobiotics). Microbial genomics shows that evolution is subject to random changes rather than governed by natural selection with the goal of increasing fitness. Indeed, stochastic and catastrophic elements can substantially reduce a population and leave only a few survivors. The proportion of those survivors can be so low that it is difficult to imagine that their survival is due to anything other than chance. For example, during a plane crash, the chances of a passenger surviving are not improved by any particular inherent genetic advantage. Population bottlenecks are an indiscriminate sampling process, and genetic drift is independent of positive selection (Figure 1). In the same way that the sampling of colored balls from an urn is not influenced by the color difference among the balls, the effect of the gene is irrelevant to evolution. In summary, even beneficial adaptations may be permanently eliminated by bottlenecks. The immediate effect of a population bottleneck is decreased genetic diversity. In the long-term, repeated population bottlenecks and the accumulation of deleterious alleles through random genetic drift in small populations can negatively affect their fitness (Ohta, 1973).

## Improvement and complexity

**Figure 2:**
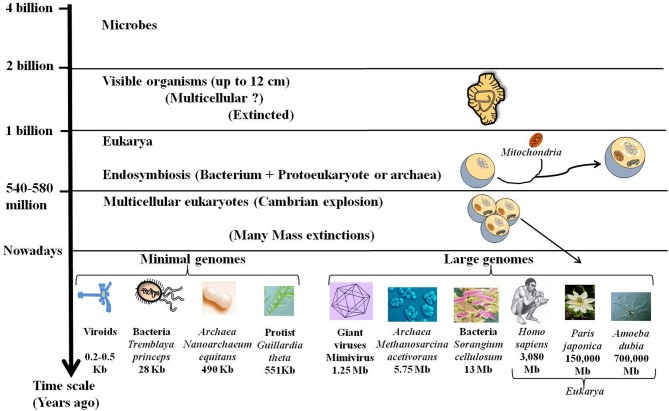
**Fossils, genome size, and complexity**. Stratified evolution as deduced from fossils discoveries is characterized by the apparition and extinction of many organisms. Nowadays some complex organisms have disappeared while others are still present. These complex organisms with large genomes including giant viruses and large eukarya coexist with simple organisms like bacteria and viroids that are able to live and multiply despite their tiny genomes. The lack of correlation of genome size with organismal complexity confirms the C-value paradox.

Darwin proposed a mechanism for the transformation of random variation into adapted, elaborate and complex devices that perform highly specific functions and increase the fitness of their carriers. Accordingly, the complex organisms, especially the multicellular eukaryotes (exhibiting large families of paralogous genes, the complicated regulation of gene expression, alternative splicing, and other genomic attributes) were considered more advanced and more successful than the simpler microbial organisms. However, genomics shows that the history of life is not a uniform trend for increasing complexity (Lynch, 2006). While, most eukaryotic taxa seem to have followed the route of junk recruitment, leading to complex organisms (Koonin, 2011c), different lineages, particularly in bacteria with an obligatory intracellular lifestyle, followed the route of genomic streamlining (Andersson and Kurland, 1998). Moreover, it has been assumed that organized multicellular life appeared with the “Cambrian explosion” some 600 million years ago. Interestingly, a group of 2.1-billion-year-old fossilized organisms (up to 12 cm) was recently found in Gabon (El Albani et al., 2010). This new discovery indicates that some large living things disappeared despite their size and complexity (Figure 2). Ultimately, genome size of present species has been revealed to be especially diverse across the different domains of life, ranging 1000-fold in viruses, bacteria and *Archaea* and 1,000,000-fold in eukarya as for the protists (Figure 2). This large diversity shows that evolution does not follow a unidirectional route towards increasing complexity.

One surprising outcome of analyzing the genome size is the lack of apparent correlation between the genome sizes and genetic and/or morphological complexity. This is the “C-value paradox,” the C-value being a measure of genome size, typically expressed in base pairs of DNA per haploid genome (Thomas, 1971; Gregory, 2005). The C-value paradox implies that organisms with similar complexity may have very different genome sizes and conversely organisms with similar C-values may not be equally complex. Thus, the organism with the largest genome [and the largest number of open reading frames (ORFs)] is not necessarily the most complex. For example, the flagellated protist *Trichomonas vaginalis* has a genome of 160 Mb with ~60,000 protein-coding genes and many repetitive regions (up to 65% of the genome) (Carlton et al., 2007). Humans and mice have a genome size of around 3 billion base pairs whereas the unicellular protozoan *Amoeba dubia* has a genome size of around 700 billion base pairs, about 200 times as big (Figure 2). Indeed, genome size and number of genes cannot be used as a predictor of genetic or morphological complexity. While, the trend towards genome streamlining led to miniaturized genomes with a high-density of protein-coding DNA, most of DNA of large genomes is non-coding. The increase in gene number in multicellular species is accompanied by an expansion in the size and number of intragenic spacers and a proliferation of mobile genetic elements. The persistence of all of these sequences in the complex genomes may be due to an inefficient purifying selection in relation to the population size (Lynch, 2007). Genome complexity seems to be an indirect consequence of reduced effective population sizes accompanied by an increase in organism size. Therefore, the evolution of genome complexity may be a non-adaptive process that occurs in response to a reduction in the population size (Lynch and Conery, 2003).

Genomics is in total disagreement with the idea that species progress toward greater complexity and increasing fitness. Indeed, the notion of success and advances can be revisited in light of microbial genomic data. The compactness of microbial genomes and their widespread abundance in the biosphere highlight the power and competitiveness of simple and streamlined genomes (Koonin, 2011c). The most common bacteria on earth *Pelagibacter ubique* has a small genome (1.31 Mb). Likewise, the delta agent virus has a single genome of approximately 1700 bp and is capable of multiplying (Hughes et al., 2011) and viroids with genetic sequences of 150–500 bases of RNA represent the simplest known elements of life and are also able to spread, multiply, and cause diseases. Moreover, the most specialized bacterial species, those with an obligate intracellular lifestyle, are the most effective at a given time and in a given ecosystem, yet, they have lost their ability to adapt outside the host cell. This loss of adaptability is likely the cost of conserving a gene pool, especially in terms of translation, that slows multiplication. Therefore, by specializing in response to particular conditions, these microorganisms lose their ability to adapt to ecosystem changes. Bacteria in an optimized system are no longer able to adapt to other systems. This principle has been described by Pasteur for immunization. Indeed, the adaptation of *Pasteurella multocida* (the agent of fowl cholera) to a new ecosystem (axenic medium) resulted in the loss of their ability to multiply in their former ecosystem (chickens) (Pasteur, 1880).

The modern study of biology has shown that random processes result in perpetual change and ongoing evolution. As Heraclitus stated, nothing in the world, even for a moment, remain identical to itself: everything passes, everything changes, and everything dies every moment (Barnes, 1983). The term “evolution” implies progress; however, the observed changes do not necessarily correspond to an optimization. Recently, along with many other critics, Cathy Cox has proposed replacing the word evolution with the term “biological changes over time,” which is a much more precise definition of the reality (http://www.georgiaencyclopedia.org/nge/Article.jsp?id=h-2622). This redefinition has resulted in major conflicts. Many biologists believe that these alternative terms exclude the idea of positive change and progress. In contrast, according to Gould, this progress is an illusion and is only a subjective interpretation of the statistics (Gould, 1984).

In practice, there is no goal of “progress” or “evolution” behind these biological changes. Some organisms are moving toward greater simplicity, whereas others become more complex without a general direction of evolution. Finally, the increasing morphological complexity does not go hand in hand with the gene repertoire complexity or with the increasing ability of adaption.

## Gradualism

Darwin thought of evolution as a gradual accumulation of small changes. This proposal is a major component and one of the most controversial of Darwin's theories. He repeated several times the Latin phrase “natura non-falcit saltum” (nature does not make laps). The punctuationists believe that all are species have their own history, appearing and then disappearing, whereas gradualists consider species with much less interest, as a concept of convenience. Like Lamarck, Darwin believed that species changed gradually by undergoing changes and modifications over time without sharp changes. Because the evolutionarily relevant mutations are supposed to have infinitesimally small fitness effects, the Darwinian model of evolution inevitably leads to the concept of gradual progressive improvement (Darwin, 1859). This vision comes from his early training as a geologist who intentionally ignored disasters and catastrophic events in evolution. We know that this view is false in both geology and biology. Rather than small, gradual changes, massive events occur that affect living beings. Thus, because a gene must be present or absent to produce an inherited effect, Mendel assumed that the appearance of a new function would occur at once rather than gradually, as Darwin imagined. Later the zoologist Ernst Mayr showed that new species generally appears in geographic isolation and undergo a true “revolution” that rapidly transforms their gene pool. Studies on the frequency and geographical distribution of chronological horse fossils show that species evolution is not linear but consist of periods of stasis (gradual changes) interspersed with “crises,” which lead to sudden extinctions and the appearance of new species. Indeed, different species could coexist with their original species while that ancestor remained unchanged, and there have even been reversals in evolutionary characteristics. These are all different evolutionary phenomena that explain the diversity of fossils and constitute a direct rebuttal to the principle of gradualism.

**Figure 3:**
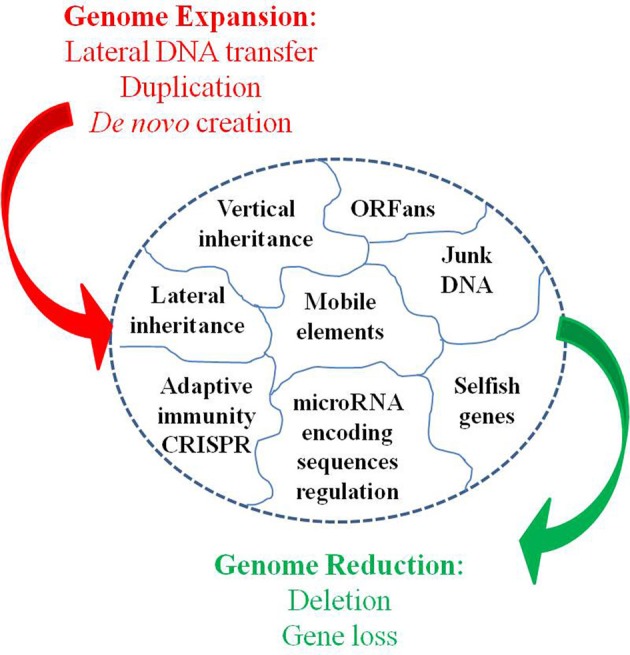
**Dynamic entity of the bacterial genomes**.

Moreover, Darwin's principle has been challenged by the Birth, Death, and Innovation Model of gene family evolution (Karev et al., 2002). In this model, duplication and lateral gene transfer give “birth” to new paralogous genes, “death” refers to gene elimination, and innovation corresponds to the acquisition of a new gene family via duplication and rapid evolution or via *de novo* creation. These events induce large and profound variations in genome size and gene repertoire (Figure 3). Thus, bacterial lineages that are specialized, including those with an obligatory intracellular lifestyle, show a repeated pattern of reduction in genome size through gene loss (Andersson and Kurland, 1998; Merhej et al., 2009). Bacterial genomes expand through lateral gene transfer and duplication. As a result, a considerable proportion (up to 14% of the ORFs) of most bacterial genomes consists of horizontally acquired genes (Nakamura et al., 2004). Lateral transfer allows for the acquisition of xenobiotic functions (Treangen and Rocha, 2011). Lederberg's work in microbiology showed that these alterations can be transmitted in a heritable manner (Lederberg, 1949). Plasmids of several hundred kilobases can be transferred, as can bacteriophages, in bacteria. This phenomenon also occurs in eukaryotes. The virus HHV6 can integrate into the genome of humans and be transferred to their children (Arbuckle et al., 2010; Raoult, 2011). Additionally, the entire genome of the intracellular bacterium, *Wolbachia* was found to be integrated into the genome of its host (Dunning Hotopp et al., 2007; McNulty et al., 2010). Some of these inserted sequences are transcribed within eukaryotic cells, indicating that they may be functionally relevant to the evolution of the microbe's host. Finally, bacterial genomes exhibit a significant number of paralogous genes due to duplication (Fitch, 1970), ranging from 7% in *Rickettsia conorii* to 41% in *Streptomyces coelicolor* A3 (Gevers et al., 2004). Gene duplication represents an important path to the evolution of new biological functions via neo-functionalization (Ohno, 1970; Innan and Kondrashov, 2010). Clearly, loss, the lateral acquisition of genes, and the emergence of a new gene as a result of duplication or *de novo* creation are far from being “infinitesimal” changes, and if such large events occur, they are too abrupt so that the gradualist paradigm is not valid.

## Vertical inheritance

Darwin believed inheritance to be strictly vertical. In contrast Lamarck believed that the adaptation of a species to an ecosystem results in the acquisition of transferable characters. Like the “infective heredity” described by Lederberg, Lamarck insisted on the “inheritance of acquired characteristics”(Koonin and Wolf, 2009). Indeed, an organic modification acquired by an individual is genetically transmitted to offspring. In contrast, Darwin thought that traits were acquired by chance and not influenced by the environment. Natural selection retains the favorable changes *a posteriori*. This view has been challenged in a number of cases. First, the theory of “use it or lose it” holds that when genes are not used in a given ecosystem, they disappear (Moran, 2002). In this case, the phenotype precedes the genotype. Second, genomics have revealed the lateral acquisition of immunity in relation to the environment rather than by chance or vertically. Indeed, clustered regularly interspaced short palindromic repeats (CRISPRs) are found in the genomes of bacteria and archaea (Grissa et al., 2007; Horvath and Barrangou, 2010). These short sequences are stored in-between repeated elements; they function as acquired immunity genes against viruses (Weinberger et al., 2012). They can be transmitted to offspring allowing them to fight against the infection of viruses that have infected their ancestor in the past. Third, the high level of the transmission of sequences between organisms is particularly remarkable for the transmission of antibiotic resistance sequences between microorganisms. During the administration of a certain antibiotic, the sequence encoding for antibiotic resistance genes amplify by recombination or by duplication or by activating the expression and spread among different microorganisms. Moreover, some antibiotics may induce generalized transduction and help to propagate resistance genes (Rolain et al., 2011). The fourth challenge to vertical inheritance is the chimerical aspect of genomes, which will be developed in the next chapter. Finally, the vertical descent theory ignores the phenomenon of increase copy number and spread of repetitive DNA elements, like the selfish genes in a dynamic that usually has little or no benefit to the fitness of the organism.

**Figure 4:**
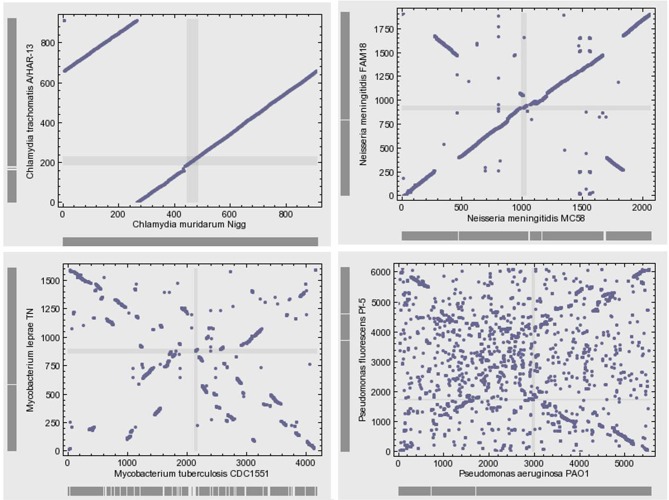
**Gene position plots of pairs of 4 selected genome pairs of bacteria show large variation between related species**. This dynamic view of the genome rejects the evolution by infinitesimal variation of vertical inheritance. Each dot represents a pair of orthologous genes identified using the bidirectional best hit approach.

Microbial genomes are not simply bags of faithfully inherited genes from an ancestor; rather, they are varied in their organization (Huynen and Bork, 1998). Bacterial genomes often exhibit high levels of plasticity and high levels of gene gain and loss during the evolution of species and strains. The genomes of closely related bacteria with different lifestyles showed remarkable variability with respect to gene content and gene order (Perna et al., 2001; Edwards et al., 2002). The microbial genomic architecture, or the arrangement of genes in a genome, exhibits great evolutionary instability (Koonin, 2009b). With the exception of the organization of small groups of functionally linked genes in operons, there is relatively little conservation of gene order, even among closely related organisms (Koonin, 2011a) (Figure 4). Various elementary mechanisms underlie the substantially dynamic character of genome evolution. Indeed, genome rearrangements such as inversions and translocations profoundly destroy the conservation of gene order. Moreover, recombination frequently occurs and generates sequence diversity by incorporating short DNA fragments (Feil et al., 2000; Hanage et al., 2005).

Comparative genomics shows large diversity in the gene repertoires among and within species. The genomes of obligate intracellular bacteria contain a subset of the genes present in their ancestors' larger genomes as the result of reductive evolution and gene loss (Merhej et al., 2009). The degree of genome flexibility correlates with the genomic content of repeated and mobile sequences such as insertion sequence elements, plasmids and phages (Mira et al., 2002). The differential gene repertoires among closely related species is most likely due to gene transfer (Perna et al., 2001; Zhang et al., 2007) and recombination of repeated sequences (Tamas et al., 2002) rather than strict vertical inheritance and random variation as Darwin suggested. A substantial fraction of the differences in gene content is due to gene duplication (Zhang et al., 2007) and to the presence of ORFans, ORFs with no detectable sequence similarity to any other sequence in the databases (Fischer and Eisenberg, 1999); they correspond consequently to hypothetical or putative proteins. It has been proposed that the majority of ORFans are derived from bacteriophages (Daubin and Ochman, 2004). The variation in gene content often yields large pan-genomes (Tettelin et al., 2005; Schoen et al., 2008). Thus, the pan-genome of the genus *Streptococcus* likely exceeds by at least three times the average genome size of a typical *Streptococcus* species (Lefebure and Stanhope, 2007). The relationship between the pan-genome of a taxon and the gene content of a specific genome in the same taxon is far from being simple (Figure 4).

Lateral transfer has been viewed as a marginal phenomenon except for the transfer of pathogenicity islands (Perna et al., 2001; Juhas et al., 2009) and antibiotic resistance (Brisson-Noel et al., 1988; Shoemaker et al., 2001; Barlow, 2009). However, the analysis of multiple complete genome sequences became feasible, lateral transfer was revealed to play a major role in the evolution of microbial organisms (Lawrence and Retchless, 2009) especially by contributing to the metabolic innovation (Ochman et al., 2000). Oxygenic photosynthesis seems to have spread by lateral transfer (Mulkidjanian et al., 2006) via bacteriophages (Lindell et al., 2005). Thus, lateral transfer plays a major role in the biochemical diversity of microbial organisms and allows them to make up the vast majority of living cells on the planet and to be the principal agents in the biosphere. In contrast to the complexity hypothesis, genomics has shown that no gene is completely refractory to lateral transfer (Jain et al., 1999; Wellner et al., 2007). It was previously thought that informational genes were less prone to lateral transfer, but genomic analysis showed that genes essential for transcription and translation had also experienced multiple lateral transfers (Brochier et al., 2000; Merhej et al., 2011). Lateral transfer affects the functions of genes to different extents, and all genes are susceptible to lateral transfer because the mechanism of transfer is random (Hao and Golding, 2008; Merhej et al., 2011). Moreover, lateral inheritance does not cleanly move a gene that is defined by start and end codons but rather involves DNA sequences that can be non-coding or include a single gene or a block of genes. It would thus be more accurate to talk of lateral sequence transfer (LST) than lateral gene transfer. Finally, the length of the lateral transfer can vary from a few bases (recombination) to multiple kilobases (Chan et al., 2009; Merhej et al., 2011). The analysis of the genomic sequence of *Wolbachia* demonstrates that LST occurs independently of the length of the sequence. Indeed, a small proportion and nearly the entire genome of *Wolbachia* in some cases, was found to be integrated into the genome of its host (Dunning Hotopp et al., 2007; McNulty et al., 2010).

Comparative genomics have shown that bacterial genomes are extremely heterogeneous and dynamic entities. The striking diversity in gene content and the flexibility of the genomic architecture challenge the theory of strict vertical acquisition and, to a greater extent the concept of species.

## Common ancestor

Darwin theorized that all extant life forms originated from a unique ancestor, which is now commonly referred to as the LUCA (last universal common ancestor). Koonin's seminal book vindicated Darwin's conjecture on the common origin of life and discussed the reconstruction of the gene repertoire of the LUCA (Koonin, 2011b). Indeed, comparative genomics revealed the universal conservation of hundreds of genes that are involved in gene expression and are thereby evidence in support of a common ancestral heritage (Koonin, 2003; Mirkin et al., 2003). The universally conserved features include the genetic code, i.e., the 64 codons that encode 20 amino acids and the stop signals; the three core subunits of the RNA polymerase; and the translation machinery composed of approximately 30 tRNAs, several translation factors, 18 amino-acyl-tRNA synthetases, and tRNA modification enzymes. Thus, by comparing the genes that present-day organisms have in common, evolutionary genomics indicate that the LUCA was a cellular organism with complete translation machinery, a core transcription system, and several metabolic pathways that included the genes required for purine and pyrimidine nucleotide biosynthesis.

The reconstruction of this ancestral cell is not plausible, because although the ancestor is primitive, its gene repertoire lacks key components that are essential for life (Mirkin et al., 2003). In particular, it is missing the genes necessary for DNA replication. Moreover, the idea of a common origin for all living beings faces substantial difficulties, including the lack of homology in the core DNA replication system components and the distinct enzymes required for lipid membrane biosynthesis in archaea and bacteria (Leipe et al., 1999; Pereto et al., 2004). As for the replication system, it has been hypothesized that the LUCA contained an RNA genome. The replacement of the RNA genome with a DNA genome and the appearance of the corresponding molecule systems would have occurred independently in the three domains of life—archaea, bacteria, and eukarya—after their divergence. Thus, the replication system was thought to have evolved in three distinct DNA viruses (prior to the existence of the DNA cell) and then transferred to the three life domains (Forterre, 1999, 2006). Another scenario is that a LUCA with a DNA genome underwent a subsequent replacement of its DNA-replication systems by non-homologous counterparts in the bacterial, archaeal, and eukaryotic lineages (Forterre, 2002). Finally, it has been suggested that a non-cellular LUCAS (last universal common ancestral state) existed as a pool of virus-like genetic elements in which the cellular key components originated. Archaea and bacteria might have independently emerged from the LUCAS, likely with numerous life forms now extinct (Koonin, 2009c). An alternative scenario postulates that the LUCA was a complex, protoeukaryotic lineage with an RNA genome present in a metabolically and morphologically heterogeneous community that gave rise to bacteria and archaea through differential gene loss (Glansdorff et al., 2008).

Multiple scenarios have been proposed to explain the origin of living beings. Regardless of which scenario is the most accurate, it has become obvious that the large diversity among species cannot be logically explained only by mutations that occurred on a unique ancestral genome (“descent with modification”). Likewise, the idea of a single mating pair at the origin of all human beings present on earth today cannot be accepted (Raoult, 2011). Several geneticists agree that “Eve” was not the only woman to conceive children who are ancestors of the current human population. Human evolution appears to be much more chimerical. Add to this, the theory of endosymbiosis showed that mitochondria were of bacterial origin from a species closely related to the Rickettsiales. Darwins reluctantly allowed for the principle of endosymbiosis but limited it to a single event suggesting that the exception does not undermine the principle of a common ancestor. However, we recently demonstrated that mitochondria were not the result of a single event but rather resulted from multiple events of gene transfer from different sources, leading to variation among organisms (Georgiades and Raoult, 2011). Mitochondria seem to have different bacterial origins, which are mainly, but not exclusively, from the group of Rickettsiales. Similarly, human beings are chimeras that contain retroviral DNA and many genes of bacterial and archaeal origin (Raoult, 2011). Genes from *Trypanosoma cruzi* are likely to integrate into the genome of infected patients and to be passed on to children according to infective heredity. Finally, giant viruses were shown to be chimeras composed of the genes of viral, bacterial, archaeal, and eukaryotic origins (Boyer et al., 2009). The notion of common ancestry completely undermines the existence of chimeras. Chimerism seems to give a clearer view of genome evolution than does common ancestry.

The hypothesis of a LUCA as a living organism with a ribosome has never been demonstrated. Livings have been classified into three domains commonly known as the Bacteria, the Archae, and Eukaryotes on the basis of ribosomal RNA sequences (Woese et al., 1990; Pace, 2006); viruses were excluded from this classification because they do not seem to possess a core of genes related pathogenicity and they lack ribosomes (Moreira and Lopez-Garcia, 2009). The idea of defining the livings based on the analysis of ribosomal genes implies that all genes are derived from a ribosome-containing organism. However, metagenomic studies that test all of the sequences in an environment show that only 15% of the sequences identified in these conditions can be linked to a cell with a ribosome. These sequences have different origins, some are viral, and others are of unknown origin. These last sequences may be either from viruses that have not yet been identified or genes that were created *de novo* (ORFans). In the other hand, the core genome of nucleocytoplasmic large DNA viruses was shown to be as ancient as the other domains of life (Boyer et al., 2010; Colson et al., 2012). Thus, asserting that life began with the existence of a ribosome and is defined by this (Moreira and Lopez-Garcia, 2009) is a form of neo-creationism. Indeed, Woese and Crick (Woese, 1967, 1970; Crick et al., 1976; Andersson and Kurland, 1990) proposed that translation started long before the ribosome creation. The initial synthesis of polypeptides did not require the elaborate machinery of ribosomes, activating factors, and enzymes, but was rather accomplished using only RNA messenger and a few primitive tRNAs. This confirms that there was life before the ribosome-containing “LUCA.” Therefore, current cells with ribosomes have incorporated sequences from viruses, newly created genes and sequences predating the ribosome apparition. All these data are contradicting the LUCA theory of a single ancestor of all currently living organisms.

Given what we know about microbiology, a scenario based on the theory of punctuated equilibrium is more likely than the Darwinian phyletic gradualism. According to Gould, long periods of relative evolutionary stability, called “stasis,” are interrupted by evolutionary changes that occur relatively rapidly (Eldredge and Gould, 1972; Gould, 2002). Some chaotic changes, such as, geological catastrophes, can be destructive steps that create a bottleneck with few survivors. It is likely that during the evolution of life there was a catastrophic event that created a bottleneck, and the surviving cells had a ribosome and, potentially, a repertoire of ancestral genes other than those encoding the ribosome, particularly the genes encoding for RNA polymerase. The selection process resulting from the bottleneck is completely random and is not influenced by the genes that may confer a greater likelihood of survival in the ecosystem. Survival of a disaster may not confer further evolutionary advantages and can in no way be regarded as natural selection of the fittest. Rather, this process is a non-directional selection without an adaptive goal; is merely chance. Migration from the area of a bottleneck gives rise to increased diversity and the creation of new species. Heterogeneous populations result from the accumulation of mutations and LST. From time to time, a stochastic event may create a new stage and induce the proliferation of a species in an ecosystem. Thus, the capacity of specialized bacteria to multiply is linked to a limited number of events; one event that seems to be particularly important is the limitation of translation capabilities. Indeed, in at least seven bacterial phyla, the evolutionary history of specialized bacteria seems to begin with the disappearance or the malfunction of the ribosomal operon, which forced the bacterium to specialize while limiting its production only to useful proteins. This change allows the specialized bacteria to expand more rapidly than others in their specific niche (Merhej et al., 2009). However, the gene repertoires of living beings did not completely disappear but some have been used and are present in a certain number of organisms that exist today. Thus, genes have an evolutionary history that is different from that of the whole organism, as postulated by Dawkins (2006).

**Figure 5:**
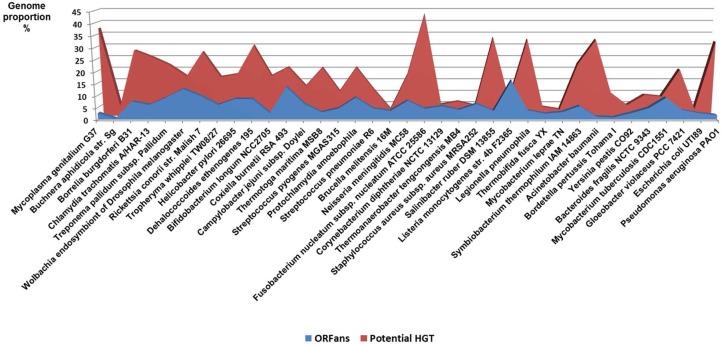
**Proportion of the ORFans and potential lateral transfer in bacterial genomes**.

Our hypothesis is that ancestral organisms were sorted by successive disasters, and some of them were able to improve their ability to live in the ecosystems in which they now live. These species represent chimeras made by combining ancestral genes with laterally acquired sequences, a mixture of genes that have been recycled from organisms that are now extinct, and genes that were newly created (Figure 5). The idea of a unique common ancestor denies chimerism and traces the creative origin of life today to an event. Many scientists adhere to this theory and end up denying the very existence of life outside of cells with a ribosome such as, viruses that may be excluded from “life.” In contrast, we believe that life cannot be considered anything other than the expression of the language contained in genetic sequences.

## Tree of life

**Figure 6:**
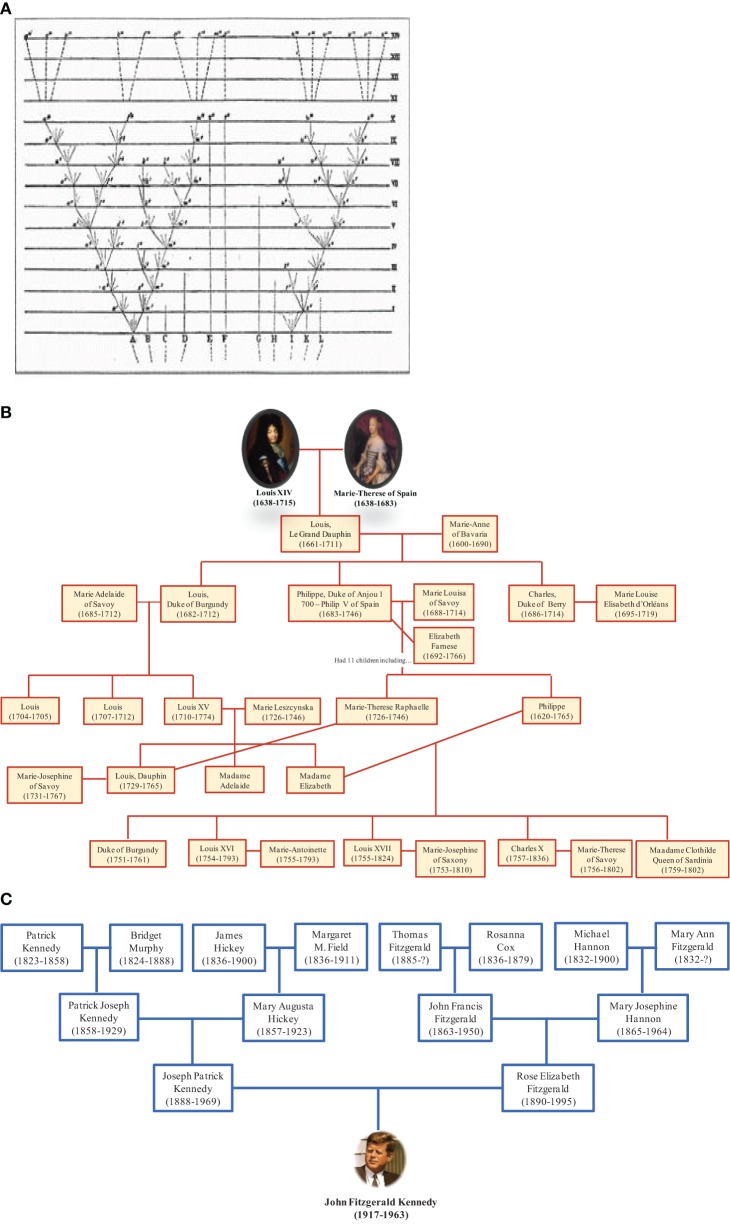
**Genealogical trees**. **(A)** *Darwin's* illustration of the origin of species in the form of a tree with lineage splitting. The trunk growing from the root split into two branches, marking the creation of two new species. The branching continued right on up to the top of the tree representing species alive nowadays. **(B)** Darwin's tree is compatible with this tree showing descendants of Louis XIV. **(C)** In contrast, the ascendant genealogy of John F. Kennedy is showing his multiple ancestors. This representation is consistent with our current knowledge.

The depiction of relationships in the form of a bifurcating tree was not invented by Darwin; it had been used for many centuries to represent descendant genealogies such as those of royal families. Likewise, the term “tree of life” (TOL) was not invented by Darwin. It is a biblical metaphor that refers to the tree of knowledge that bears the fruit that gives eternal life (Bible, Genesis 2:9 and 3:22). Darwin adopted the living tree analogy to illustrate the mechanism of evolution by showing continuity between populations and species and demonstrating that certain lineages of species compete and supplanted other lineages (Penny, 2011). Thus, Darwin represented the evolution of life as a hierarchical pattern of relationships that reflects the “natural order” (Doolittle, 1999). Darwin's TOL assumes that all life forms originated from a single node corresponding to a last common ancestor through a branching evolutionary process (Doolittle and Bapteste, 2007). From this perspective, it is interesting to see the representation of trees of life in the form of family trees. Some genealogical trees begin with an ancestor and show his descendants. It should be noted that this tree does not represent reality because we know that current human beings do not descend from a single ancestor but result from many couples, forming an inverted genealogic tree (Figure 6). These genealogical representations ignore chimerism and LST and instead show existence of our human lineage as descending from a single ancestor. People commonly understand evolution in terms of multiple species descending from a common ancestor; the reality may more closely resemble the opposite, with multiple ancestors contributing genes to individual species.

The accuracy of the common origin and the relevance of the tree-like representation as a model of evolution have been frequently questioned (Bapteste et al., 2009; Dagan and Martin, 2009; Puigbo et al., 2010). The TOL concept presumes that all organisms are descended from a predecessor. This is true for a number of genetic sequences but not for some ORFans, including the functional ORFans. Indeed, some genes and proteins have been entirely invented in the last million years. For example, genes that are specific to the species of *Drosophila*, have been demonstrated to be essential, or at least useful, for the current life of *Drosophila* (Chen et al., 2010). These genes originated in an ancestor of *Drosophila* for which they were useful, but they were never created elsewhere. The TOL is a perception of conservative nature that lost the ability to create anymore new function since the ancestor was alive. This is contrary to our current knowledge. The analysis of bacterial genomes shows that between 10 and 15% of the genes of each species has no equivalent in other species and are likely due to “gene creativity (Figure 5).” Some of these genes may have been created by the reconstruction of old genes or by the genetic drift of unused genes, resulting in useful features that persevered, while other genes disappeared. This demonstrates the constant creative trial that enables the creation of new life forms.

**Figure 7:**
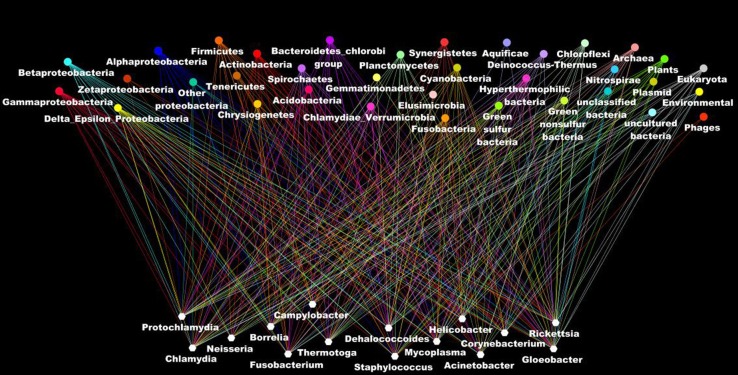
**The ascendant genealogy or the rhizome of bacteria**. Bacterial genomes (at the bottom in blank) have a mosaic structure as a result of lateral inheritance from the different organisms in the different taxonomic group (at the top of the figure). Each line indicates the taxonomic origin of the putative closest phylogenetic organism as deduced from the BLastP analysis of all the genes in the genomes.

Other evidence that deeply undermines the TOL is LST. Indeed, single gene phylogenies often yielded conflicting topologies that are distinct from the rRNA phylogenetic tree (Maddison, 1997). The causes of these discrepancies can be analytical such as limitations of the models of amino acid sequence evolution, taxon sampling, and selection bias (Rokas et al., 2003). Nevertheless, it has been stated that beyond the analytical limitations, the evolution of genes is rather reticulate due to lateral DNA transfer, and the history of life cannot be properly represented by bifurcating trees (Doolittle, 1999). Indeed, microbial genomes contain multiple selfish elements, such as bacteriophages, gene transfer agents (Paul, 2008), plasmids, and transposable elements, that are known as the mobilome. They are involved in the lateral transfer of their associated genes via different mechanisms, including conjugation, transduction, and transformation (Frost et al., 2005; Thomas and Nielsen, 2005; Asadulghani et al., 2009). Comparative genomic and phylogenetic analyses have provided evidence of extensive LST (Ochman et al., 2000; Gogarten et al., 2002; Boucher et al., 2003; Gogarten and Townsend, 2005). Thus, hyperthermophilic bacteria were found to exhibit much higher sequence similarity to the archaea that share the same habitat than to mesophilic bacteria, likely as the result of archaea-to-bacteria LST (Aravind et al., 1998; Nelson et al., 1999). Likewise, our analysis of 16 bacterial genomes found a significant proportion of genes without homologs in closely related species but with homologs in distantly related taxa (Figure 5). These genes were likely acquired through lateral transfer. Evidence of LST according to a sympatric model of evolution is present in obligate intracellular bacteria that share the same host (Moliner et al., 2010; Coscolla et al., 2011; Georgiades et al., 2011; Merhej et al., 2011). The high prevalence of LST raised the notion of a connected microbial “gene pool” with no barrier (Beiko et al., 2005; Koonin, 2011d) while questioning the concept of bacterial species (Bapteste et al., 2009). Moreover, the dynamic nature of evolution, in which the genetic information of living organisms is inherited not only vertically but also laterally, challenges the representation of the evolution of life in the form of a Darwinian bifurcating tree (Bapteste et al., 2004, 2005; O'Malley and Boucher, 2005; Jeffroy et al., 2006; Susko et al., 2006; Marttinen et al., 2012) (Figure 7).

The fluidity of microbial genomes has instigated many efforts to find a better representation of the dynamic relationships that shape microbial evolution. It has been proposed that congruent topologies of trees for several highly conserved genes might better represent the history of the majority of the genes (Wolf et al., 2002; Dagan and Martin, 2006). Using a comprehensive comparison of individual gene tree topologies, the “forest of life” (FOL), a collection of phylogenetic trees for all genes, was proposed as an alternative to a single tree (Puigbo et al., 2009; Koonin et al., 2011). In this approach, the topologies of the 102 nearly universal trees (NUTs) were highly consistent and seemed to represent a central evolutionary trend in the FOL. The consensus topology of the NUTs has been proposed as an accurate representation of the evolution of organisms. For other scientists, the dynamic picture of the prokaryotic world is best represented as a complex network of genetic elements that exchange genes. Considering the high level of horizontal inheritance, microbial evolution more closely resembles a rhizome than a bifurcating tree (Raoult, 2010; Merhej et al., 2011; Ramulu et al., 2012) and the tree-like representation should be completely abandoned in favor of a web-like representation of evolution (Sneath, 1975; Gogarten et al., 2002; Doolittle and Bapteste, 2007; Puigbo et al., 2010; Popa et al., 2011). Unlike the hierarchical tree-like model, the novel representations that consider the broad-spectrum of gene origins, including vertical descent, lateral inheritance, and *de novo* creation, are promising representations of microbial genome evolution.

## Conclusions

**Figure 8:**
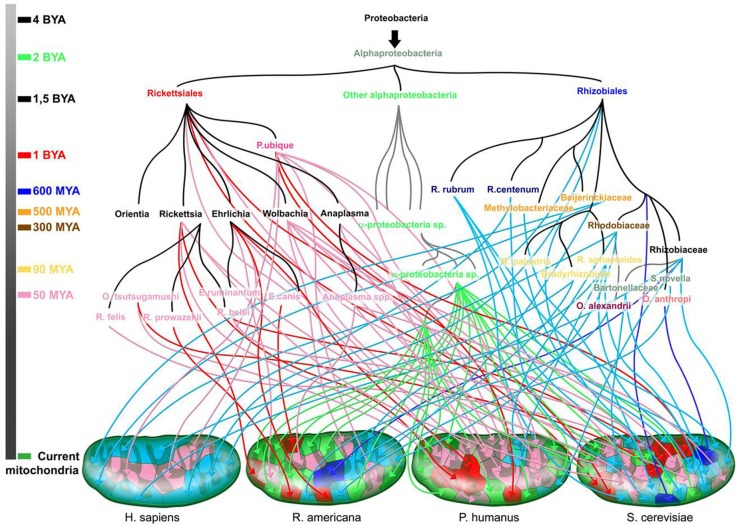
**The rhizome of mitochondria (adapted from Georgiades and Raoult (2011)**. The origin of each gene is represented along with the time scale of the species divergence. Dark blue and red arrows are for sister taxa with high bootstrap values, and light blue and pink arrows for sister taxa with low bootstrap values. Green arrows are for sister taxa from the Alphaproteobacteria subgroup. Colors on the time scale coincide with the emergence of the corresponding colored species.

None of the seven points laid out in the introduction to this manuscript can be permanently retained, as established by Darwin's theory, which was at the time a fight against the creationists. This theory cannot be upheld in its entirety. Recent advances from genomics refute the ideas of gradualism, exclusive vertical inheritance, evolution selecting the fittest, a common ancestor and the TOL. Indeed, there may not be any two genes that have the same evolutionary tree. Moreover, it is less the genes that are traded than the sequences themselves. Genes may have portions of sequences with different evolutionary origins because of recombination. An accurate representation of the genealogy of genes in a repertoire should take into account the different origins of closely and distantly related organisms as well as organisms that have gone extinct. A single tree is largely inadequate. We prefer to represent evolution as a family tree or in the form of a rhizome, which corresponds to a more authentic description of our present knowledge than the TOL (Figure 8).

Finally Darwin has contributed to the debate on the myth from the Bible and Aristotle and tried to return the history of life to the domain of science. At the same time, he created a cultural and religious context, a sort of scientific battle against obscuring belief. Indeed, he is considered in Britain and the United States of America as an icon of science against the obscurantist religious or the creationists (Raoult, 2008, 2010). The expression of Darwinian's idolatry peaked in the year 2009 which corresponded to the bicentenary of his birth and the 150th year of his theory, when virtually all scientific journals posted photos and texts on Darwin. Currently, even in the USA, the opinion is divided on evolution at about equal between evolutionists and creationists. This position has become ideological so that many of the major writers of the twentieth century in the field of evolution felt compelled to take a stand on the issue. Mayr, Gould, and Dawkins stated theories that are antagonist to that of Darwin. Moreover, Karl Popper claimed that the theory of evolution was not a scientific theory (Popper, 2002). From our point of view, the theory of evolution is a scientific theory however it is an outdated theory. Darwin's theory should not become a religion but remain a scientific theory from another era that can be refined based on the actual insights from microbial genomics.

### Conflict of interest statement

The authors declare that the research was conducted in the absence of any commercial or financial relationships that could be construed as a potential conflict of interest.
